# Osteocyte morphology and orientation in relation to strain in the jaw bone

**DOI:** 10.1038/s41368-017-0007-5

**Published:** 2018-02-26

**Authors:** Vivian Wu, René F. M. van Oers, Engelbert A. J. M. Schulten, Marco N. Helder, Rommel G. Bacabac, Jenneke Klein-Nulend

**Affiliations:** 1 Department of Oral Cell Biology, Academic Centre for Dentistry Amsterdam (ACTA), Amsterdam Movement Sciences, University of Amsterdam and Vrije Universiteit Amsterdam, Amsterdam, The Netherlands; 2 Department of Dental Materials Science, Academic Centre for Dentistry Amsterdam (ACTA), Amsterdam Movement Sciences, University of Amsterdam and Vrije Universiteit Amsterdam, Amsterdam, The Netherlands; 3 Department of Oral and Maxillofacial Surgery, VU University Medical Center/Academic Centre for Dentistry Amsterdam (ACTA), Amsterdam Movement Sciences, Amsterdam, The Netherlands; 4 Department of Physics, Medical Biophysics Group, University of San Carlos, Cebu City, Philippines

## Abstract

Bone mass is important for dental implant success and is regulated by mechanoresponsive osteocytes. We aimed to investigate the relationship between the levels and orientation of tensile strain and morphology and orientation of osteocytes at different dental implant positions in the maxillary bone. Bone biopsies were retrieved from eight patients who underwent maxillary sinus-floor elevation with β-tricalcium phosphate prior to implant placement. Gap versus free-ending locations were compared using 1) a three-dimensional finite-element model of the maxilla to predict the tensile strain magnitude and direction and 2) histology and histomorphometric analyses. The finite-element model predicted larger, differently directed tensile strains in the gap versus free-ending locations. The mean percentage of mineralised residual native-tissue volume, osteocyte number (mean ± standard deviations: 97 ± 40/region-of-interest), and osteocyte shape (~90% elongated, ~10% round) were similar for both locations. However, the osteocyte surface area was 1.5-times larger in the gap than in the free-ending locations, and the elongated osteocytes in these locations were more cranially caudally oriented. In conclusion, significant differences in the osteocyte surface area and orientation seem to exist locally in the maxillary bone, which may be related to the tensile strain magnitude and orientation. This might reflect local differences in the osteocyte mechanosensitivity and bone quality, suggesting differences in dental implant success based on the location in the maxilla.

## Introduction

Bone quality at the patient’s implant site is an important local factor for the success of dental implants^1,2^. It influences the dental implant stability at the time of surgery^3,4^ and osseointegration at the second stage^5,6^. Implant success rates are different at various implant sites in the jaw, with the highest failure rates occurring in the maxillary posterior region^7^. Bone quality is determined by mechanical properties as well as the bone mineral density, bone architecture, and extracellular matrix composition^1,8^.

Bone structure is continuously remodelled by bone-resorbing osteoclasts and bone-forming osteoblasts, which are regulated by osteocytes^9^. Osteocytes act as mechanosensors of bone producing signalling molecules that affect osteoblastic and/or osteoclastic activities. A prominent theory is that mechanosensing by osteocytes occurs via strain-induced fluid flow through the lacuno-canalicular network^10^.

Osteocyte morphology varies in different types of bone. Elongated osteocytes are found in load-bearing long bones that are predominantly loaded parallel to their longitudinal direction. On the other hand, round osteocytes are found in flat bones, such as calvariae, loaded with much lower amplitudes, radially and/or tangentially, due to intracranial pressure and/or mastication^11^. Osteocyte morphology and orientation thus seem to be affected by the mechanical loading direction. Osteocyte lacunae are aligned to the collagen fibre orientation^12,13^, which may correspond to the orientation of the tensile strain in the bone^14^. External mechanical forces on cells are known to affect the cytoskeletal structure and thus the cell morphology^9,15^. Moreover, round osteocytes are much more mechanosensitive than elongated cells^16^. Round osteocytes in calvarial bone experience much lower mechanical loads than long bones, which might indicate that their morphologies maintain their physiological functions even in the presence of low mechanical loads and hence are more mechanosensitive than elongated osteocytes in long bones that are exposed to higher mechanical loads^11^. Therefore, the osteocyte morphology at the implant location may predict the success of dental implants.

Bone quality is assessed by bone density in bone biopsies using histomorphometry and densitometry, but the cellular parameters for bone quality have not been determined^17^. To date, not much is known about the osteocyte morphology and orientation in the human jaw bone.

In this study, it is hypothesised that 1) tensile strains in the maxillary bone are larger and more uniformly directed in a single gap compared to free-ending locations and 2) osteocytes are larger, more elongated, and more uniformly oriented in a single gap versus free-ending locations. Therefore, this study aimed to investigate the relationship between the levels and orientation of tensile strain and the morphology and orientation of osteocytes in single gap versus free-ending dental implant positions in the maxillary bone through a finite element (FE) model and histomorphometric analyses.

## Results

**Fig. 1:**
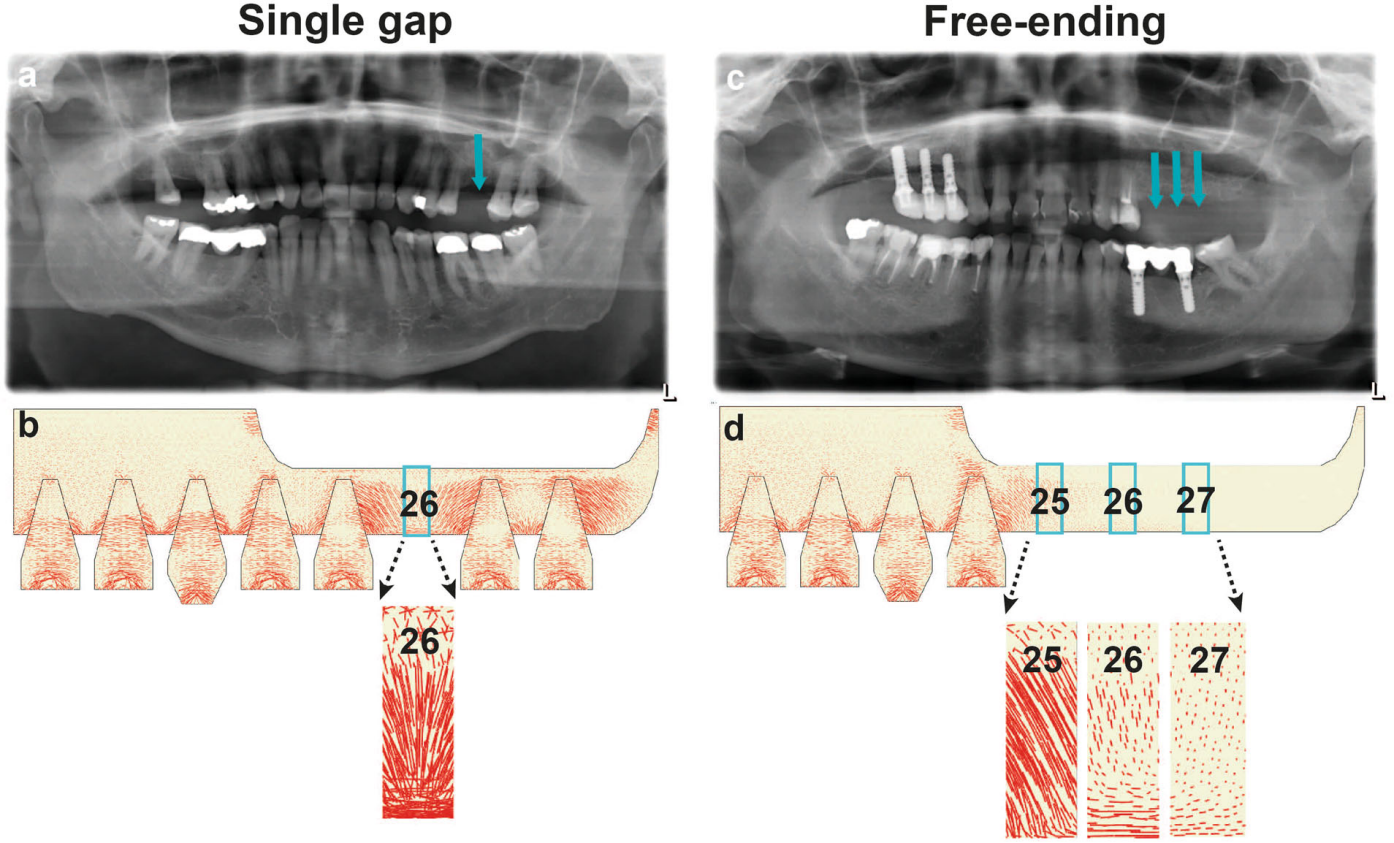
Panoramic radiograph and finite element model representing the single gap and free-ending locations in the maxillary bone prior to biopsy retrieval. **a** Radiograph showing the single gap location prior to biopsy retrieval (26), directly between two neighbouring natural teeth. **b** FE model predicting the large tensile strain in the single gap location (26). A ×7.5 magnification of the tensile strain shows the tensile strain directed from the natural tooth to the single gap location. **c** Radiograph showing the free-ending locations prior to biopsy retrieval (25, 26, 27). There are no natural teeth distal from the biopsy locations present. **d** FE model showing a decreasing tensile strain magnitude with the distance from the natural tooth; the location (27) experienced the smallest tensile strain compared to the other locations (25, 26). A ×7.5 magnification of the tensile strain showing the tensile strain oriented from the mesial neighbouring natural tooth to the free-ending locations (25, 26, 27). The red lines indicate the tensile strain in the maxillary bone; the direction and length of the red lines indicate, respectively, the direction and magnitude of the tensile strain in the maxillary bone

**Fig. 2:**
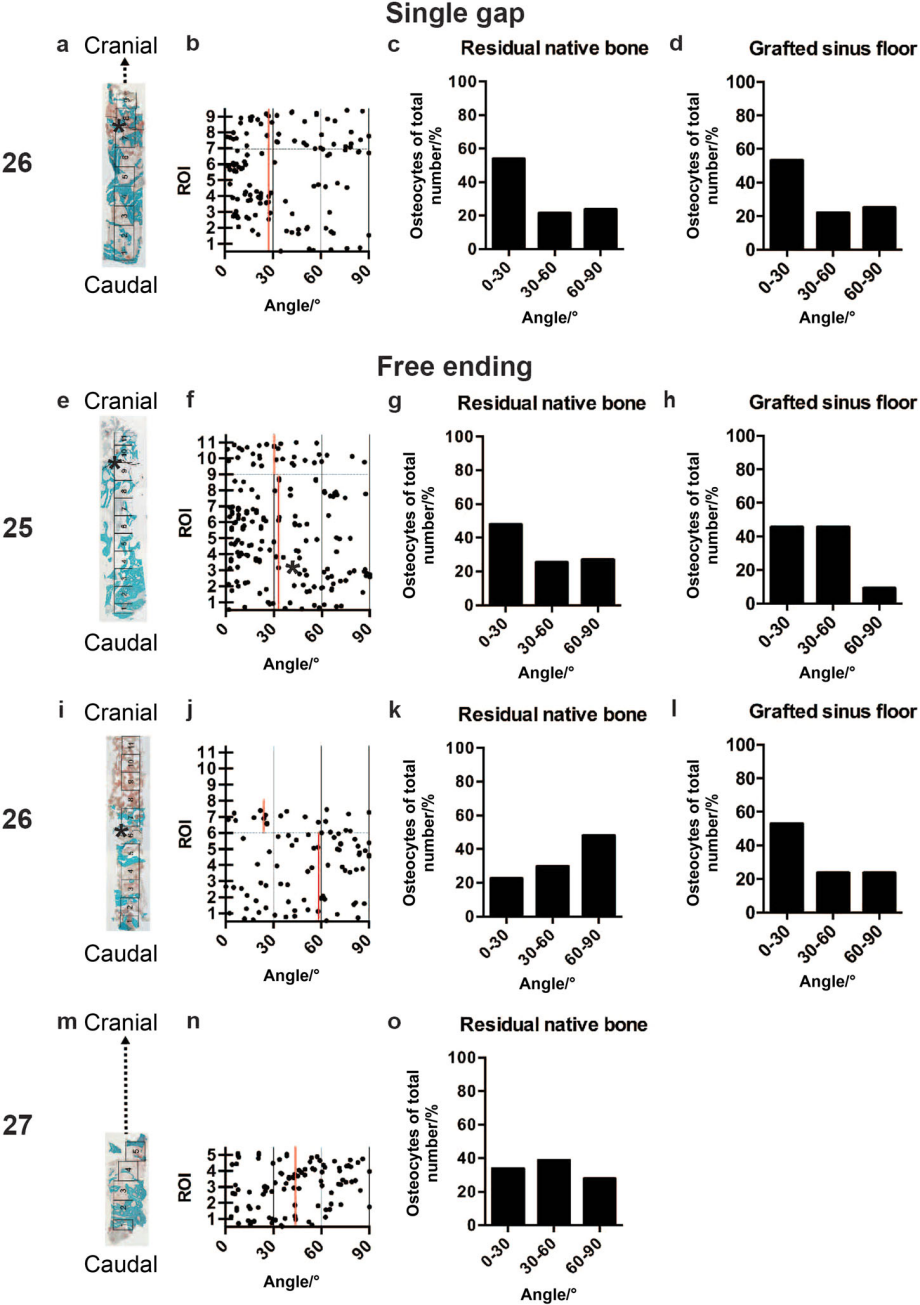
Histology and osteocyte orientation in the maxillary bone. **a** Single gap location (26). Overview of a mid-sagittal section of the whole biopsy stained with Goldner’s Trichome. For histomorphometrical analysis, the biopsy was divided in consecutive ROIs of 1 mm^2^. The maxillary sinus floor indicates the border between the residual native bone (RNB) and the grafted maxillary sinus floor (GMSF) (*). Mineralised bone tissue (green) and unmineralized osteoid (red) were both observed in the RNB and GMSF. Some part of the biopsy was broken and excluded from the histomorphometric analysis (dotted lines). **b** Schematic overview of the osteocyte orientation per ROI. Every dot in the diagram represents a measured osteocyte. The vertical red line indicates the median of the osteocyte orientation. The horizontal black dotted line represents the border between the ROIs from the RNB and GMSF. **c** Evaluation of the osteocyte orientation in the RNB at the single gap location (26). **d** Evaluation of the osteocyte orientation in the GMSF at the single gap location (26). **e** Free-ending location (25). Mineralised bone tissue and unmineralized osteoid were both observed in the RNB and GMSF. **f** See the description for **b**. **g** Evaluation of the osteocyte orientation in the RNB at the free-ending location (25). **h** Evaluation of the osteocyte orientation in the GMSF at the free-ending location (25). **i** Free-ending location (26). Mineralised bone tissue was only observed in the RNB, and unmineralized osteoid was found in in RNB and GMSF. **j** See the description for **b**. **k** Evaluation of the osteocyte orientation in the RNB at the free-ending location (26). **l** Evaluation of osteocyte orientation in the GMSF at the free-ending location (26). **m** Free-ending location (27). Mineralised bone tissue and unmineralized osteoid were both observed in the RNB. Some part of the biopsy was broken and excluded from the histomorphometric analysis (dotted lines). **n** See the description for **b**. **o** Evaluation of the osteocyte orientation in the RNB at the free-ending location (26). ROI, region of interest

The maxillary bone biopsy details and histomorphometrical analysis results of the residual native bone (RNB) data in the eight patients are shown in Table 1. The evaluation results of two patients, a single gap location (Patient #1) and free-ending locations (Patient #5), are shown in detail in Figs 1 and 2.

**Table 1:** Maxillary bone biopsy details and histomorphometrical analysis of residual native bone (RNB)

Patient number	Biopsy location	Dental implant position	Time/months	Bone class	*N*	*R*/%	Area mean ± standard deviations/µm^2^	Orient/%		
								0°–30°	30°–60°	60°–90°
1	Single gap	26	8	II	15	8.8	78.5 ± 31.1	54.8	21.5	23.7
2	Single gap	26	9	II	70	8.3	56.0 ± 25.8	40.0	30.8	29.2
3	Multiple gap	16	> 36	III	58	7.4	25.6 ± 12.2^a^	37.0	31.5	31.5
		17	13	III	71	13.2	65.2 ± 28.7	47.6	33.3	19.1
4	Multiple Gap	24	11	II	83	7.8	45.8 ± 25.6^a^	41.6	49.3	9.1
		25	11	II	67	3.5	43.6 ± 20.4^a^	35.9	35.9	28.2
		26	9	II	121	4.3	35.1 ± 22.0^a^	16.4	38.8	44.8 *
								
5	Free-ending	25	> 36	III	151	11.6	46.4 ± 32.0^a^	47.8	25.4	26.8
		26	10	III	78	12.0	57.9 ± 23.4^a^	22.5	29.6	47.9 *
								
		27	6	III	108	11.1	47.6 ± 26.6^a^	33.3	37.5	29.2
6	Free-ending	23	3	III	108	6.5	57.5 ± 34.8^a^	57.4	23.8	18.8
		25	> 24	III	119	19.0	59.5 ± 30.2^a^	55.0	30.0	15.0
		26	9	III	34	9.5	31.2 ± 13.1^a^	35.7	32.1	32.1
7	Free-ending	24	12	III	226^a^	8.0	53.3 ± 21.8^a^	50.0	32.5	16.7
		26	12	III	146	22.6	69.5 ± 35.6	43.0	34.4	22.6
8	Free-ending	25	> 24	III	184	7.5	72.3 ± 32.7	30.7	29.7	39.6 *
		26	10	III	51	13.5	87.9 ± 54.4			
								28.9	37.8	33.3 *
								

Osteocyte number, morphology (round versus elongated), surface area and orientation were assessed.

^a^Significantly different from the average of patient #1 and #2, *P < 0.05.

Time, Time of biopsy retrieval after tooth extraction (month); Bone class, Clinical bone quality classification (Lekholm and Zarb^18^); N, number of osteocytes per region of interest; R, round osteocytes; Area, osteocyte surface area; Orient, osteocyte orientation

### Patients

The period between the tooth extraction and biopsy retrieval at the single gap location was eight to nine months (Table 1: Patient #1 and #2). While this period at the multiple gap locations varied from nine to > 36 months (Table 1: Patient #3 and #4), this period spanned three to > 36 months in the free ending locations (Table 1: Patient #5 – #8).

Preoperatively, the clinical bone quality was classified as type II in all of the single gap locations and as type III in all of the free-ending locations (Table 1: Bone class)^18^. Clinical bone quality did not seem to be directly correlated with the period between the tooth extraction and biopsy retrieval. No clinical signs of inflammation were observed during biopsy retrieval.

### Radiological evaluation

Panoramic radiographs were made preoperatively (not shown) and six months after maxillary sinus floor elevation (MSFE) prior to biopsy retrieval. A single gap location in patient #1 (Fig. 1a) and free-ending locations in patient #5 (Fig. 1c) are shown. The mean height gain of the maxillary sinus floor at the biopsy positions was similar for all patients (mean ± standard deviations: 7.5 mm ± 1.7 mm).

### FE model

**Fig. 3:**
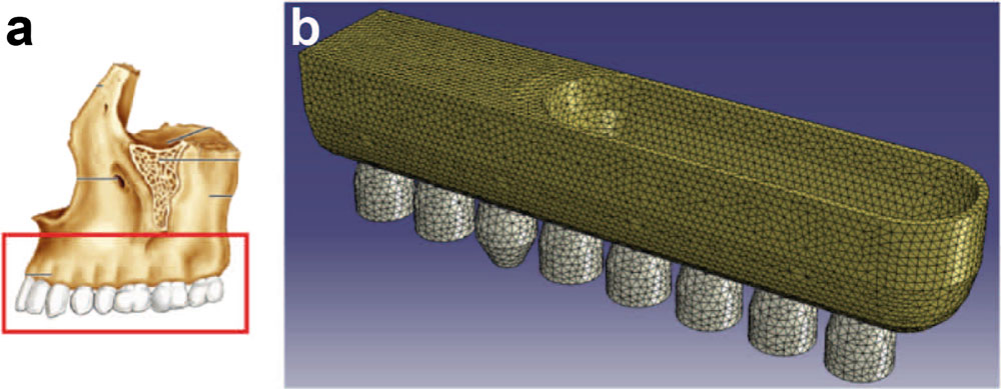
Translation of the maxillary dental arch into an finite element model. **a** Anatomical image of the maxillary bone showing one side of the superior dental arch (adapted from Marieb and Hoehn (2014)^28^. **b** The FE model showing the maxillary dental arch with the sinus cavity. Individual teeth can be removed from this model to simulate patient-specific cases

The FE model predicted that the tensile strains were the largest close to the natural tooth and decreased in magnitude with increasing distance from the tooth. This strain profile was caused by the bending of the sinus floor between the mesial and distal teeth. To better understand the strain profile, it should be kept in mind that the respective figures show a 2D section from a 3D model (Fig. 3). In the 3D model, the maxillary sinus floor was also supported by the buccal and lingual sides of the sinus cavity.

The tensile strain magnitude and direction were different in the single gap and free-ending implant locations. In the single gap locations, the tensile strain was large and uniformly directed in the cranial-caudal direction (Fig. 1b). In the free-ending locations, the tensile strain magnitude decreased >2-fold by one tooth distance from the natural tooth (Fig. 1d). The tensile strain was less uniformly oriented than in the single gap locations.

### Histology

All of the intact biopsies and a few broken but well evaluable biopsies were included in the study. Newly formed mineralised bone tissue, containing lacunae with live osteocytes, unmineralized osteoid areas, and connective tissue were observed around the β-tricalcium phosphate (β-TCP) particles cranial to the native residual bone in the biopsies. Approximately 10% of the observed lacunae were empty in all of the regions. Bone ingrowth was determined from the border between the RNB and the grafted maxillary sinus floor (GMSF) towards the cranial side. The newly formed bone was in close contact with the bone substitute granules (Fig. 2a, e, l, m). Some biopsies showed no newly formed mineralised bone tissue, but only unmineralized osteoid areas and connective tissue in the most cranially located region of interests (ROIs) (Fig. 2i).

### Histomorphometrical evaluation of the mineralised residual native bone tissue

No differences were observed in the mean percentage of the mineralised native residual bone tissue volume between the single gap and free-ending implant locations (mean ± standard deviations (%); single gap: 33.4 ± 14.1; free-ending: 32.8 ± 20.4, 27.3 ± 13.9, 37.4 ± 19.7).

### Osteocyte number and morphology in the residual native bone

The single gap, multiple gap and free-ending implant locations showed similar numbers of osteocytes per ROI (mean ± standard deviations: 97 ± 40) (Table 1). In all locations, most of the osteocytes (~90%) were elongated, while only ~10% of cells were round (Table 1). One free-ending location (24) had significantly more round cells than other locations (Table 1; Patient #7). In a single gap location (26), the osteocyte surface area was ~1.5 times larger than in the multiple gap and free-ending locations (Table 1; Patient #1–6) (*P* < 0.05).

### Osteocyte orientation

The osteocyte orientation in the single gap locations of two patients was similar (Table 1: Patient #1 and #2). As a reference value, the average osteocyte orientation of patient #1 and #2 was taken for the other multiple gap and free-ending locations. The osteocyte orientations of a single gap location patient #1 and a free-ending location patient #5 are shown in detail (Fig. 2).

The osteocyte orientation was similar in the single gap (26; patient #1) RNB (median: 27.4°) and GMSF (median: 27.4°) (Fig. 2b). In the RNB, 54.8% of osteocytes had a cranial-caudal orientation (Fig. 2c), and in the GMSF, 53.1% of osteocytes had a cranial-caudal orientation (Fig. 2d).

The osteocyte orientation was similar in the free-ending (25; patient #5) RNB (median: 32.9°) and GMSF (median: 30.3°) (Fig. 2f). A total of 47.8% of the osteocytes in the RNB had a cranial-caudal orientation (Fig. 2g), and 45.5% of the osteocytes in the GMSF had a cranial-caudal orientation (Fig. 2h). Moreover, the osteocyte orientation was different in the free-ending location (26) between the RNB (median 58.0°) and GMSF (median: 25.8°) (Fig. 2j) (*P* < 0.05). A total of 22.5% of the osteocytes in the RNB had a cranial-caudal orientation (Fig. 2k), and 52.9% of the osteocytes in the GMSF had a cranial-caudal orientation (Fig. 2l). The osteocyte orientation in the free-ending location (27) was only measured in the RNB (median 43.2) since the GMSF was lacking (Fig. 2n). A total of 33.3% of the osteocytes had a cranial-caudal orientation (Fig. 2o). The osteocyte orientation in the RNB in the free-ending location (26) was different from the single gap reference value (Table 1: Patient #5) (*P* < 0.05).

Moreover, the osteocyte orientations in the free-ending (25; patient #8) and (26; patient #8) RNB were significantly different from the reference values of patient #1 and #2.

## Discussion

The aim of this study was to investigate the relationship between the tensile strain and osteocyte morphology and orientation in human maxillary bone. An FE analysis and histological and histomorphometrical data were used to predict the possible differences in the maxillary bone quality between the single gap versus free-ending locations. The FE model predicted larger and differently oriented tensile strains in the single gap compared to free-ending implant locations. Histomorphometrically, no differences were observed for the mineralised RNB volume and the number and morphology of the osteocytes between single gap and free-ending locations. The osteocytes in the single gap locations had a more cranial-caudal orientation and a larger surface area than in the free-ending locations. These results suggest possible differences in the dental implant success related to the osteocyte mechanosensitivity in the single gap and free-ending implant positions in the maxilla.

Although FE modelling is used extensively to predict the biomechanical stress directions in dental implants and its surrounding bone in relationship to implant success^19^, it has never been used in relationship to osteocyte morphology and orientation. The presence of the remaining teeth near the implant position keeps the bone mechanically strained. The tensile strains were directed from the natural tooth to the biopsy location(s), resulting in a difference in the tensile strain orientation between the single gap and free-ending locations.

In the FE model, individual teeth were removed to simulate patient-specific cases. Whereas the biopsies showed a heterogeneous patchwork of cortical bone, trabecular bone, β-TCP granules, and connective tissue, the sinus floor was modelled as a homogeneous tissue of intermediate stiffness. This was done for two reasons: (i) there were not sufficient 3D data present for each patient to model the actual heterogeneity of the whole sinus floor and (ii) the focus was on the broad stress trajectories resulting from the remaining dentition, which was best investigated by leaving the other factors equal.

Even though the time of extraction appeared to be comparable (8–13 months) for most of the retrieved biopsies, there was a clinical difference in the bone quality between the single gap versus free-ending locations, which was class II in most of the gap locations and class III in the free-ending locations. This suggests a higher amount of cortical bone and lower amount of cancellous bone in the single gap than in the free-ending locations. However, no differences were observed between the mineralised bone tissue volumes between the different locations, suggesting no changes in bone formation at the time of biopsy retrieval. Since osteocytes fulfil a role as mechanosensors of bone, it is plausible that bone formation is affected differently in the single gap than in the free-ending implant positions in the long term due to differences in the tensile strain magnitude and orientation.

Nearly all osteocytes were elongated in both the single gap and free-ending locations, implying a dominant loading direction in these bone regions. Osteocytes in the gap locations showed significantly larger surface areas than those in the free-ending locations, suggesting differences in osteocyte mechanosensitivity. Since the osteocyte cell body likely plays a role in direct mechanosensing of the matrix stiffness, this might relate to differences in bone architecture^16,20^. Moreover, it has been shown in vitro that different mechanical stimuli cause different cellular deformations^21^. This would suggest that differences in tensile strain result in changes in the osteocyte cytoskeleton and different morphologies^9^. Since differences in osteocyte morphology were not observed, the differences in the tensile strain might have been too small to cause substantial cytoskeletal changes.

Osteocytes in the single gap locations and free-ending locations directly neighbouring a natural tooth on one side had a cranial-caudal orientation, resulting from large and uniformly directed tensile strain. The osteocytes in the various free-ending locations of one patient had different orientations from each other and the single gap location, resulting from a decrease in the tensile strain magnitude from the natural tooth to the most distal free-ending location. These data are in line with previous observations showing elongated osteocytes aligned in the principal loading direction and osteocytes aligned to the collagen fibre orientation^12,13^, which corresponds to the orientation of the tensile strain in the bone^14^.

A limitation of our study was that we only used two-dimensional sections to analyse the orientation of the three-dimensional osteocytes. However, this did not affect our conclusion regarding any possible differences in the morphology between the different implant locations since histomorphometry is based on the principle that statistical information of three-dimensional structures can be obtained from two-dimensional cross-sections if a sufficient number of cross sections is measured. Information regarding surfaces can be obtained from cross-sections of these surfaces, i.e., lines. Another limitation of this study is the small number of patients.

In conclusion, these data show significant differences in the surface area and orientation of osteocytes, in particular, in the areas of the maxillary bone that are related to the tensile strain magnitude and orientation. The exact implication of the osteocyte orientation on the dental implant success, however, is complex and deserves further study. This exploratory study provides, for the first time, a view on the relationship between tensile strain and the osteocyte morphology and orientation in the maxillary bone, which might contribute to a better understanding of the cellular processes that lead to different bone qualities in various dental implant positions and, eventually, to the success of dental implants in the maxilla.

## Materials and methods

### Patient selection

Eight patients, six men and two women, who were partially edentulous in the posterior maxilla, were selected. All patients required a MSFE due to insufficient maxillary bone height, and the vertical bone height before MSFE was 4–10 mm.

The mean age of patients was 58 years (range: 40–73 years). All patients were non-smokers or smoked <10 cigarettes per day. Patients with systemic diseases, drug abuse, and/or pregnancy were excluded from participation, as well as patients requiring horizontal bone augmentation.

The study was performed in accordance with the principles of the Declaration of Helsinki. Since the study involved CE-marked calcium phosphates being used for their intended purpose (carrier material for bone augmentation in MSFE procedures), no specific regulatory approval from a medical ethical committee was required. Patients provided written informed consent before inclusion in the study.

### Clinical bone quality classification

The bone quality was pre-operatively assessed and was classified based on the amount of cortical bone versus cancellous bone^18^.

### Maxillary sinus floor elevation surgery

A preoperative panoramic radiograph was made from each patient to calculate the alveolar bone height at each planned implant position. The MSFE procedure was performed with Ceros® β-TCP granules with 60% porosity/0.7–1.4 mm grain size (Thommen Medical AG, Grenchen, Switzerland) as previously described^22^. The oral mucoperiosteal flap was closed using Gore-Tex sutures (W.L. Gore and Associates, Newark, DE, USA), which were removed 10–14 days post-operatively. All patients received antibiotic prophylaxis consisting of 500 mg of amoxicillin 4-times daily, starting one day preoperatively and continuing for one week postoperatively. After a healing period of six months, prior to dental implant placement, a panoramic radiograph was made to measure the available tissue height for the dental implant placement. The dental implants were placed as previously described^23^.

### Biopsy retrieval

Bone biopsies were collected using a trephine burr (outer and inner diameter 3.5 mm and 2.5 mm, respectively) and were fixed in 4% phosphate-buffered formaldehyde (Klinipath BV, Duiven, The Netherlands). Subsequently, the biopsies were transferred to 70% ethanol and were stored until use for histomorphometry. One midsagittal section per biopsy was evaluated as described below.

Seventeen biopsies from gap or free-ending locations were evaluated. The following dental implant location definitions were used: 1) single gap location: a natural tooth is present at both sides of the implant location; 2) multiple gap location: a natural tooth is present on either side of at least two implant locations next to each other, and multiple bone biopsies can be retrieved in this gap; and 3) free-ending location: there is only one natural tooth present at one side (mesially) of the implant location(s), and multiple bone biopsies can be retrieved in this situation.

### FE model

A three-dimensional model of the maxillary sinus floor (Fig. 3) was designed with the FE software Abaqus/CAE (version 6.12, Dassault Systemes Simulia, Providence, RI, USA) to predict the tensile strain in the maxillary bone. Individual teeth were removed from this model to simulate patient-specific cases. While the dimensions of the model approximate the anatomical dimensions, the dental arch was straightened to simplify the visualisation and for comparison with the dental radiographs.

All material behaviour was assumed to be linear-elastic and isotropic. The maxillary bone was modelled with a Young’s modulus of 10 GPa and Poisson’s ratio of 0.3. The cortical and cancellous bone were not distinguished, since the demarcation between these two bone types varied greatly in MSFE patients. The teeth were given a Young’s modulus of 24 GPa, which was based on the stiffness of dentin. The focus was on the strains at the implant positions rather than within the remaining teeth, and therefore, the enamel and pulp were not modelled as separate materials.

Teeth were loaded with an occlusal (vertical) load of 100 N, which is comparable to the human bite force^24^. As a boundary condition, the bone section was fixed in the mesio-distal direction at its mesial surface and was fixed in all directions at its cranial surface.

### Histology and histomorphometrical analysis

After dehydration in an ascending alcohol series, the bone specimens were embedded without prior decalcification in low temperature polymerising methylmethacrylate (MMA, Merck Schuchardt OHG, Hohenbrunn, Germany) as previously described^25^. Longitudinal sections of 5 μm thickness were prepared, and the midsagittal sections were stained with Goldner’s Trichome to distinguish mineralised bone tissue (green) and unmineralized osteoid (red)^26^.

The histological sections were divided into ROI of 1 mm^2^. Each ROI was analysed separately using a Leica DC 200 digital camera and Leica QWin^©^ software (Leica Microsystems Image Solutions, Rijswijk, The Netherlands) as well as ImageJ (US National Institutes of Health, Bethesda, MD, 1997–2014). A demarcation line indicated the transition from the RNB floor to the regenerated GMSF bone.

Mineralised bone tissue was calculated as the mean percentage of the mineralised volume in each ROI as previously described^22,27^.

### Osteocyte morphology and orientation

**Fig. 4:**
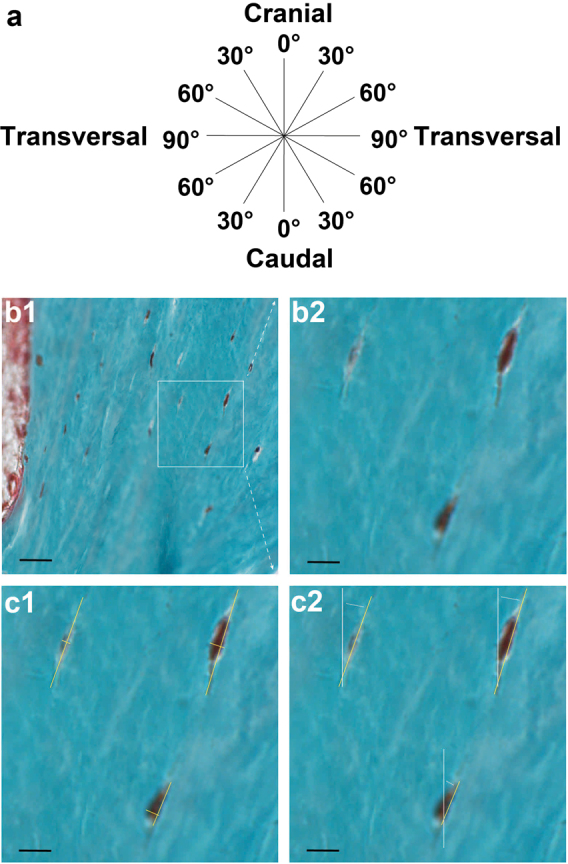
Histomorphometrical analysis of the osteocyte surface area and orientation in biopsies from the maxillary bone. **a** Schematic diagram showing the osteocyte orientation in the maxillary bone described by an angle from 0°–90° measured from the cranial-caudal axis. **b1** Digital image showing an overview of a random bone area in an ROI at ×400 magnification depicting osteocytes with lacunae (brown), mineralised bone tissue (green), and unmineralized osteoid (red). Scale bar, 25 µm. **b2** Digital image showing a × 1 322 magnification of the area indicated in B1. Scale bar, 7.5 µm. **c1** The yellow lines on the osteocyte represent the measured length and width of the osteocyte. The length was determined as the longest distance (length axis) of the cell, and the width (width axis) was determined as the longest distance that meets the length axis at a right angle. The osteocyte surface area was computed by the ellipse formula “*π* × ½ length × ½ width” for a square measure. The osteocyte morphology was determined by the formula “width/length”; “< 0.8,” indicating elongated and “≥ 0.8,” indicating round. Scale bar, 7.5 µm. **c2** The orientation of the osteocyte was measured by the angle between the length axis of the osteocyte (yellow line) and the cranial-caudal axis of the biopsy (white line). The white dotted line shows the angle measured between the two axes. Scale bar, 7.5 µm. ROI, region of interest

The osteocyte morphology and orientation were analysed in each ROI (Fig. 4). In a random bone area in each ROI, a digital image was acquired at ×400 magnification (Fig. 4b1). Only sharp and clearly displayed lacunae with live osteocytes were analysed. The osteocyte number, surface area, morphology, and orientation were determined “blindly” by two researchers.

For the osteocyte surface area calculation, the ellipse formula “*π* × ½ length × ½ width” was used (Fig. 4c1). Osteocyte morphology was defined with the formula “width/length”; “ < 0.8” distinguishing elongated and “≥ 0.8” distinguishing round (Fig. 4c1). The osteocyte orientation was described by an angle from 0°–90° measured from the caudal-cranial axis. The cranial-caudal axis side was labelled 0°, and the transversal axis was labelled 90°. The osteocyte orientation was measured by the angle between the length axis of the osteocyte (Fig. 4c2: yellow line) and cranial-caudal axis (Fig. 4c2: white line).

### Statistical analysis

The data are presented as the mean ± standard deviation. Statistical analyses were performed using SPSS version 20 software. The Mann–Whitney test and Pearson Chi-square test were performed to compare the results obtained from the different volumes of interest between the biopsies in a gap and free-ending implant position. Statistical significance was considered when *P* < 0.05.
